# Lightweight Geometric Framework for High-Precision 3D Gaze Tracking Based on Infrared Image Processing

**DOI:** 10.3390/s26123741

**Published:** 2026-06-12

**Authors:** Jiawei Shen, Pengxiang Dong, Beichen Hu, Yuanqing Wang

**Affiliations:** School of Electronic Science and Engineering, Nanjing University, Nanjing 210023, China; 602023230092@smail.nju.edu.cn (J.S.); 652024230026@smail.nju.edu.cn (P.D.); 502025230090@smail.nju.edu.cn (B.H.)

**Keywords:** 3D gaze tracking, geometric modeling, lightweight framework, eye tracking, infrared image processing

## Abstract

Head-mounted eye-tracking systems play a critical role in virtual reality, human–computer interaction, and clinical applications, yet achieving both high angular accuracy and precise 3D gaze position estimation with low-cost hardware remains challenging. This paper proposes a lightweight, training-free geometric 3D gaze tracking framework for binocular 3D gaze tracking using consumer-grade hardware, which leverages stereo geometric triangulation and a simplified physiological eye model to achieve robust 3D gaze estimation, requiring only standard infrared cameras and dichroic mirrors without additional specialized hardware. The method was evaluated in controlled indoor conditions with 30 participants, where it achieved an angular error ranging from 1.1° to 2.82° and a 3D gaze position error below 13.24 mm. Compared to two state-of-the-art academic non-deep-learning methods, the proposed framework delivers competitive angular accuracy while significantly reducing 3D position error, outperforming the baselines by 34% to 56% in depth estimation precision. These results demonstrates that the proposed geometric framework is a practical and effective solution for high-precision 3D gaze tracking on low-cost hardware, suitable for both research and consumer applications.

## 1. Introduction

Eye-tracking technology captures the movement of the human eye and calculates gaze direction, facilitating contactless human–computer interaction. Compared to traditional input devices, it offers the characteristics of flexibility, speed, and accuracy, enabling direct two-way information transmission between humans and machines [1,2]. Emerging fields, such as telemedicine, intelligent industries, and entertainment, require timely and efficient human–computer interaction, driving the need for versatile and precise solutions with significant application potential. Eye tracking technology has become a foundational enabler for a wide spectrum of human–computer interaction applications, spanning immersive augmented reality and virtual reality headsets, portable laptop-based interaction systems, driver monitoring platforms, and assistive technologies for individuals with motor impairments. Currently, existing gaze tracking technologies are primarily categorized into two types: screen-based gaze tracking and head-mounted gaze tracking. Screen-based gaze tracking typically employs visible light cameras to capture images of the eyes and project gaze direction onto a screen [3,4]. This method is limited to two-dimensional tracking and is restricted to fixed-position screen interactions, resulting in low accuracy and a lack of flexibility. In contrast, head-mounted gaze tracking usually utilizes infrared imaging technology for three-dimensional tracking [5,6,7]. This technology captures eye images and calculates gaze direction, offering higher accuracy and allowing for more lifelike interaction scenarios. This paper presents a self-developed, lightweight geometric framework for head-mounted binocular 3D gaze tracking, designed to deliver accurate 3D gaze vectors using only low-cost hardware without deep learning. The key contributions of this study include a robust geometric formulation that achieves competitive angular accuracy while significantly improving 3D position precision, and comprehensive evaluations comparing the proposed method with both state-of-the-art academic approaches and representative commercial systems. The remainder of this paper is structured as follows: Section 2 reviews the related work of existing gaze tracking methods. Section 3 details the system setup and the proposed geometric framework. Section 4 describes the experimental design, including the participant pool, evaluation conditions, and performance metrics, and further presents the experimental results by comparing the proposed method with both academic baselines and commercial eye-tracking systems. Finally, Section 5 concludes the paper and outlines directions for future work.

## 2. Related Work

Accurate estimation of gaze direction is fundamentally reliant on the precise extraction of pupil contour and the exact determination of pupil center position. Kagemoto et al. proposed high-speed pupil tracking using an event camera based on the bright and dark pupil effect. Two illumination sources generated events in the pupil area, and the pupil center was determined in real time at over 2000 Hz without requiring complete image from the events [8]. Also, Lu et al. proposed a valuable industry-oriented ellipse detector by arc-support line segments, which simultaneously reached high detection accuracy and efficiency. Extensive experiments on three public datasets were implemented and their method achieved the best F-measure scores compared to the state-of-the-art methods [9]. Notably, a model based on convolutional neural networks has been widely adopted in this field, enabling models to learn patterns of human eye movement from extensive datasets. Also, they proposed a novel 3D pupil localization method with a deep learning-based corneal refraction correction. Their method outperformed the state-of-the-art works by reducing the 3D pupil localization error by 47.5% and the gaze estimation error by 18.7% [10]. In addition, Xiong et al. proposed a lightweight pupil localization algorithm, which utilized a convolutional neural network (CNN) with additional training samples. The experimental results demonstrated the algorithm’s significant effectiveness in identifying the pupil position within the training set, with the accuracy of pupil position in the test set reaching 97.78% [11]. These approaches significantly improved the accuracy and robustness of gaze tracking. Furthermore, low-resolution facial images remain a major bottleneck for practical gaze tracking. To address this, Yan et al. proposed FSKT-GE, a lightweight knowledge transfer framework. It aligns intermediate features via cosine similarity, transferring high-resolution knowledge to low-resolution networks. Evaluated on Gaze360 and RT-Gene with 2×–8× downsampling, it achieves MAEs of 10.97–13.61° and 6.73–7.75°, outperforming mainstream methods [12]. Additionally, lightweight deep learning models have been optimized for gaze tracking on edge devices. Habib et al. optimized MobileNetV3 via structured pruning and post-training quantization, achieving an ultra-lightweight model for multimodal eye-gaze and emotion recognition. Their method reduces inference time to 3 ms while maintaining high accuracy, offering an efficient solution for real-time gaze estimation on resource-constrained devices [13]. However, the methods mentioned above necessitated substantial datasets for training, involved protracted development periods, and incurred high costs.

As consumer demand for seamless, intuitive, and always-on interaction grows, there is an urgent need for eye tracking solutions that deliver high 3D gaze estimation accuracy while remaining compatible with low-cost, resource-constrained, and battery-powered devices. However, existing state-of-the-art systems fail to meet this dual requirement; commercial solutions, such as Apple Vision Pro with M5 [14], VIVE Focus Vision [15], and Windows Studio [16], achieve exceptional angular accuracy but rely on specialized, high-cost hardware, closed-source proprietary algorithms, and significant computational overhead, making them prohibitively expensive and power-hungry for mass-market portable electronics.

## 3. Theory

### 3.1. Optical Path Design of the Device

**Figure 1:**
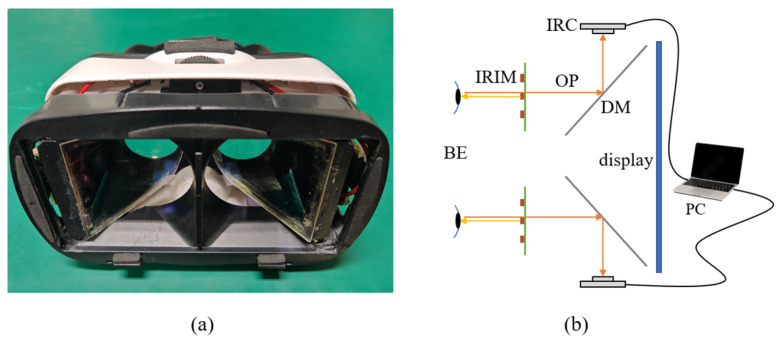
(**a**) Real object picture of the gaze tracking device. (**b**) Schematic diagram of the optical path. BE: binocular eye; IRIM: infrared illumination module; OP: optical path; DM: dichroic mirror; IRC: infrared camera; PC: personal computer. The yellow solid lines with arrows denote the incident infrared illumination path emitted from IRIM and projected onto human eyes, while the orange lines represent the reflected light from eyeballs, which is reflected by DM and captured by IRC for subsequent gaze computation on PC.

The pixel-level 3D gaze tracking system and optical path structure are depicted in Figure 1. To ensure an unobstructed view of the screen integrated into the device and to efficiently arrange the infrared camera in a confined space, the infrared camera is ingeniously positioned on the inner edge of the head-mounted device. As illustrated, a dichroic mirror is strategically placed at a 45° angle between the infrared camera and the user. This mirror reflects infrared light while allowing visible light to pass through. Adjacent to the user’s eyes, an infrared illumination panel composed of eight LED beads is carefully arranged to illuminate the eye area, facilitating image acquisition by the infrared camera. This innovative optical path design not only provides an unobstructed view for the user but also ensures the precise capture of eye images.

### 3.2. System Composition

**Figure 2:**
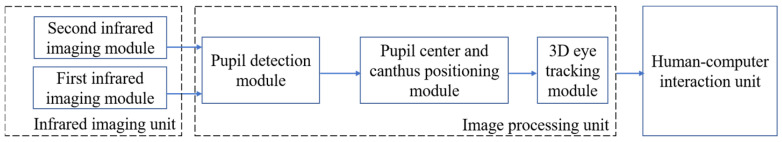
Gaze tracking system composition.

This system consists of three primary components: an infrared imaging unit, an image processing unit, and a human–computer interaction unit. The infrared imaging unit employs a binocular infrared camera to capture real-time images of human eyes, providing binocular infrared images with a resolution of 1920 × 1080 pixels. It also includes a two-phase color plate and an LED complement plate, which transmits visible light while reflecting infrared light. The LED complement plate serves to provide stable infrared illumination, thereby ensuring high-quality image acquisition. The image processing unit is responsible for pixel-level processing of the infrared images collected simultaneously from both eyes. It determines the orientation of the pupil centers, the medial canthus and the lateral canthus, and calculates the gaze vector in a spatial coordinate. The Human–Computer Interaction Unit serves as the output component of the gaze tracking system; this unit comprises a display module and a network module. These components facilitate the display of measurement results, device networking, and data transmission. The unit enhances the scalability of the gaze tracking device, allowing connectivity with production and entertainment equipment located remotely via a 5G network, thereby enabling data sharing. The composition of the system is illustrated in Figure 2.

### 3.3. Pupil Feature Extraction

**Figure 3:**
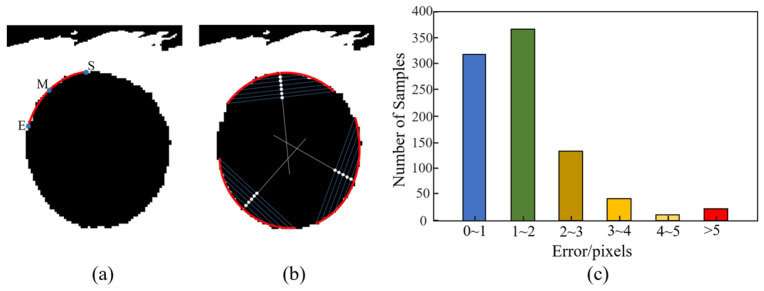
(**a**) Pupil feature extraction. The red curve denotes the contour of the pupil. Three characteristic points E (endpoint), S (endpoint), and M (midpoint) are labeled in blue dots on the arc segment. (**b**) Pupil center localization. Multiple parallel blue secant lines are sampled on the grouped arc boundary, with white dots marking the midpoints of each secant, and straight line segments connect these collinear white midpoints. (**c**) Error distribution of pupil detection.

Leveraging the dark pupil effect, the pupil region is delineated using a binarization method, as illustrated in Figure 3a. Prior to binary segmentation, bilateral filtering and histogram equalization are employed to enhance image contrast [17]. To eliminate factors such as eyelashes, lighting variations, and eye movement, potential candidate areas must be screened. First, the area size of the connected domain is calculated to identify and exclude regions that significantly differ from the expected pupil size. Next, based on the coordinates of the extreme points in all four directions of the connected domain, a bounding rectangle is constructed. The connected domain is then rotated at multiple angles, and the area of each bounding rectangle is calculated to determine the minimum bounding rectangle. Since the pupil shape is typically round or nearly round, a threshold interval for the aspect ratio is established to filter the potential pupil region effectively, facilitating accurate detection of the pupil position. Extensive analysis of the experimental data led to the establishment of a threshold range from 0.9 to 1.6.

### 3.4. Pupil Center Localization

Exploiting the circular shape of the pupil and the dark pupil effect, the pixel distribution at its edge exhibits convexity, while the gray level within the pupil is significantly lower than that outside it. As illustrated in Figure 3a, for each arc region, the midpoint and two endpoints are designated as points *M*, *S*, and *E*. If the rotational trends of points $\vec{SE}$, $\vec{SM}$, and $\vec{ME}$ are consistent, while the rotational trends of adjacent vectors fall within a specified threshold, along with the direction of the gray value gradient aligning with the vector’s rotational trend, the region will be classified into the arc group for further processing. Subsequently, several parallel secant lines are established for each arc within this group, and the midpoints of all secant lines are identified [18]. The midpoints of secant lines within the same arc are then connected by line segments, and the intersection points of these segments are determined. Using the obtained intersection positions, the pupil center is estimated through clustering, voting, and cross-verification. This algorithm effectively circumvents the computational intensity associated with traditional region growing approaches.

To rigorously evaluate the robustness of the proposed algorithm, we conducted experiments using the large-scale public iris image dataset CASIA-Iris-Lamp [19], released by the Institute of Automation, Chinese Academy of Sciences. The resolution of images within the dataset is 640 × 480 pixels; a distinctive feature of this dataset is that all images were captured under challenging conditions, including occlusions from thick eyebrows and interference from dark eyelashes. These factors enable the dataset to closely simulate various real-world disturbances that may occur during practical pupil image acquisition, providing a highly realistic foundation for assessing algorithm robustness.

From this dataset, 1000 images were randomly selected to construct a standardized test set. For manual annotation, three experienced annotators independently labeled pupil centers using the LabelMe open-source pixel-level image annotation tool. The inter-rater consistency among multiple annotators was measured by the two-way random single-measure intraclass correlation coefficient, and its computational formula is written as

(1) $$ICC=\frac{MS_{B}-MS_{E}}{MS_{B}+(k-1)MS_{E}}$$

where *MS_B_* is the mean square between subjects, *MS_E_* stands for the mean square error, and *k* represents the total number of annotators involved in manual labeling. The obtained *ICC* value reaches 0.96, proving outstanding consistency of manual annotation results. Annotation uncertainty was quantified as the average deviation among annotators, which was no more than 1.2 pixels. All annotators were blinded to the method identity to avoid bias. The proposed pupil detection algorithm was then applied to this set to estimate the pupil center for each image. Using the manually annotated labels as ground truth, we computed the mean squared error (MSE) between the algorithm’s outputs and the ground truth. The calculation formula of *MSE* is defined as

(2) $$MSE=\frac{1}{N}\sum_{i=1}^{N}\left[{\left(x_{i}-{\hat{x}}_{i}\right)}^{2}+{\left(y_{i}-{\hat{y}}_{i}\right)}^{2}\right]$$

where *N* denotes the total number of test images, (*x_i_*, *y_i_*) represents the manually annotated pupil center coordinates, and $\left({\hat{x}}_{i},{\hat{y}}_{i}\right)$ is the pupil center estimated by the proposed method. As is shown in Figure 3c, the findings indicate that the algorithm delivers strong localization performance: in more than 30% of the samples, the pupil center error remains within a single pixel, demonstrating extremely high precision. When the error tolerance is relaxed to 3 pixels—sufficient for most real-world application scenarios—the detection accuracy increases to 92.5%. To further highlight the advantages of the proposed method, we conducted comparative experiments against three mainstream pupil localization approaches under the same test conditions using a 3-pixel error tolerance as the unified evaluation criterion. The comparison methods include the traditional Hough transform, a gradient-based detection method [20], and a fully convolutional neural network (FCNN)-based pupil center detection method [21]. All baseline methods were re-implemented in-house following their original descriptions, ensuring identical preprocessing, evaluation metrics, and data splits across all methods. The results, summarized in Table 1, clearly show that under identical conditions, the proposed algorithm significantly outperforms the competing methods in localization accuracy, demonstrating its strong performance in complex scenarios.

### 3.5. Gaze Point Estimation Model

**Figure 4:**
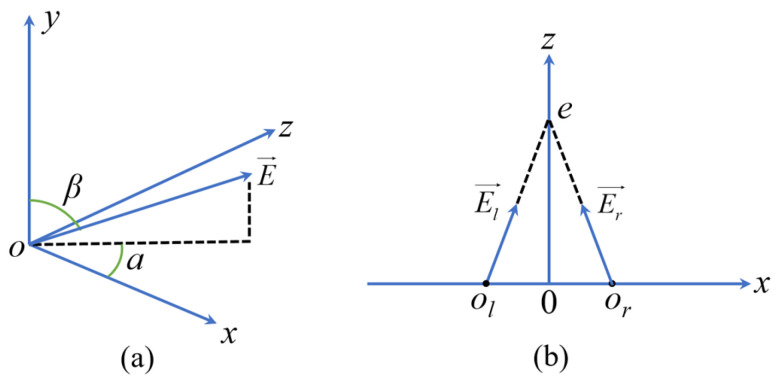
Schematic diagram of gaze vector coordinate systems. (**a**) Spherical coordinate system for single-eye gaze direction representation. (**b**) Binocular gaze geometry setup.

A spatial gaze point model is proposed to enable real-time estimation of three-dimensional gaze points from two-dimensional pupil coordinates. The method computes the precise eye-rotation angle by analyzing the relationship between pupil center displacement and eye rotation. As shown in Figure 4, the eyeball is simplified as a perfect sphere centered at point *o*. A spherical coordinate system (*r*, *α*, *β*) with its origin at the eyeball center is adopted to represent the direction of the gaze vector, where *r* is the length of parameter vector, and the midpoint between the centers of the left and right eyeballs is defined as the coordinate origin. The left and right gaze vectors are projected to intersect at the common fixation point e in three-dimensional space. Figure 4 depicts the geometric configuration of the gaze vectors.

Figure 4a shows the spherical coordinate system for single-eye gaze direction representation, where *α* denotes the azimuth angle and *β* denotes the elevation angle of the gaze vector *E*. Figure 4b depicts the binocular gaze geometry setup, with the coordinate origin set at the midpoint of the left and right eyeball centers *O_l_* and *O_r_* (separated by a distance d). The intersection of the left and right gaze vectors *E_l_* and *E_r_* forms the common 3D fixation point e. After detecting the pupil center, the key points around the eyelids are used to approximate the upper and lower eyelid boundaries, from which adjacent edge segments are extracted. Curves are then fitted to the upper and lower eyelid segments to compute curvature, and curvature extrema are identified as candidate medial and lateral canthi. The facial midline is used to distinguish the two canthi, and the candidates are further evaluated based on edge strength to determine the optimal locations. Finally, quadratic fitting of local brightness is applied to refine the coordinates. The midpoint of the line connecting the two canthi is then used as a reference point for determining the relative spatial relationship with the pupil center. Considering that the midpoint of the center line of the two eyeballs is the coordinate origin, the direction vector of the center of the pupil is *E*. In the horizontal viewing state, the direction vector of the midpoint of the line connecting the medial canthus and lateral canthus is *I*, which can be represented as

(3) $$\left\{\begin{array}{l} E=(r\sin\beta \cos\alpha ,r\sin\alpha \sin\beta ,r\cos\beta )\\ I=(r\sin\beta_{0}\cos\alpha_{0},r\sin\beta_{0}\sin\beta_{0},r\cos\beta_{0}) \end{array}\right.$$

**Figure 5:**
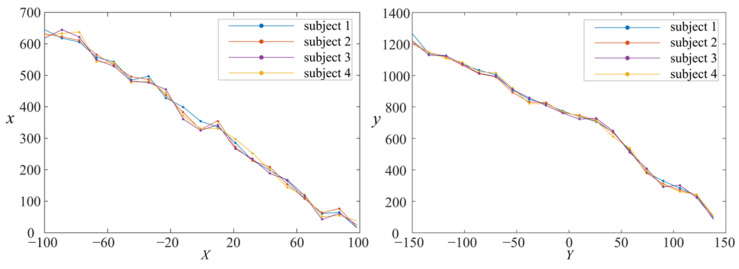
Mapping distributions between X and x, as well as between Y and y.

Given the variations in the physiological structures of the eyes among different test objects, an initialization process is necessary prior to use to ensure the accuracy of the formula parameters. The mapping of coordinate differences from an infrared image to gaze direction involves two critical components: the mapping of the user’s gaze onto the screen interface equipped with the device, and the subsequent mapping of the screen interface into three-dimensional physical space. It is important to note that the mathematical models governing both mapping components require an essential initialization process before their official operation. In order to discover the mapping relationship between *X* and *x*, as well as between *Y* and *y*, we conducted experiments on multiple users and randomly selected data of four participants, as shown in Figure 5.

It can be seen that the mapping between the pupil center and the pixel plane satisfies the following relationship:

(4) $$\begin{array}{l} x=k[X(E)-X(I)]+b_{x}\\ y=k[Y(E)-Y(I)]+b_{y} \end{array}$$

In the above equation, *x* and *y* are the horizontal and vertical coordinate values on the pixel plane respectively. In mapping information from infrared images to gaze vectors within three-dimensional physical space, we employ a mathematical model based on two spherical coordinate systems centered on the eyes. This model formulates a matrix equation of the form Ax = B, utilizing specific mathematical expressions derived from the pixel distance differences $\Delta_{x}$ and $\Delta_{y}$ in binocular infrared images, while integrating geometric relationships. The values ${∆}_{x}$ and ${∆}_{y}$ are calculated as the differences between the coordinates of the tracked gaze position and the reference point, denoted as *x* and *y*, respectively. Consequently, the following matrix expression can be further derived:

(5) $$\left(\begin{array}{cccc} y & 1 & -b_{y} & 0\\ x & 0 & -b_{x} & 1 \end{array}\right)\left(\begin{array}{c} \frac{1}{kr}\\ cos\beta_{0}\\ \frac{1}{kr}\\ sin\beta_{0}sin\beta_{0} \end{array}\right)=\left(\begin{array}{c} cos\beta \\ sin\alpha sin\beta \end{array}\right)$$

### 3.6. Initialization and Gaze Point Estimation

**Figure 6:**
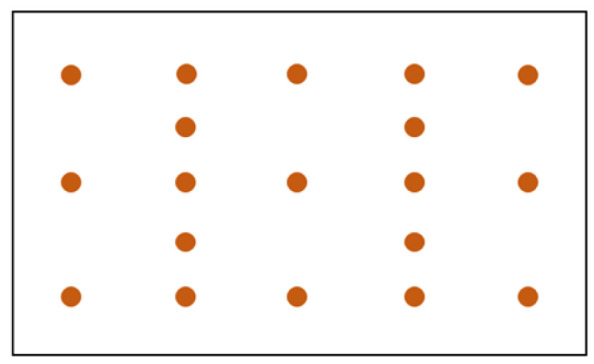
The distribution of the points on the screen.

The initialization process primarily involves two parameter modifications. Numerous prior experiments have demonstrated that the mapping from the pupil to the screen adheres to a functional relationship of a single variable, allowing the determination of the slope *k* and intercept *b* for different users within a specific range. Initially, the empirical value of *k* is provided. The user must first fixate on a designated point on the screen to calculate the value of *b*. Subsequently, the user fixates on a different point on the screen to correct the value of *k*, which ultimately leads to the formulation of the initialization function. The distribution of the points on the screen that the subjects need to gaze at to complete the initialization is shown in Figure 6.

As shown in the figure above, the black box represents the screen and the brown dots are the 19 points that need to be focused on. By gazing at points on the screen, two sets of calculated values for *k* and *b* are gathered. The root mean square (RMS) of *k* and *b* are calculated respectively for further correction, which finally yields a reliable mapping function. The expression of RMS is shown in Equation (6).

(6) $$RMS_{k}=\sqrt{\frac{1}{n}\sum_{i=1}^{n}k_{i}^{2}},RMS_{b}=\sqrt{\frac{1}{n}\sum_{i=1}^{n}b_{i}^{2}}$$

**Figure 7:**
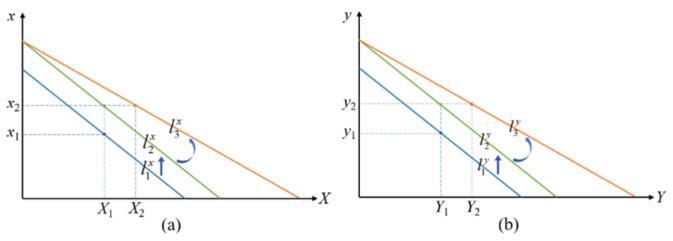
(**a**,**b**) are the evolution processes of the initialization function of x and y coordinates, respectively. The transition from line *l*_1_ to *l*_2_ corresponds to the correction of *b*, and the transition from *l*_2_ to *l*_3_ corresponds to the correction of *k*.

As shown in Equation (6), *n* represents the number of points that the subjects need to gaze at during the initialization process. It is important to note that the initialization process for mapping horizontal and vertical coordinates to points on the screen is identical. The underlying principle is illustrated in Figure 7.

The parameters for the pupil center position are stored within the three-dimensional gaze tracking module:

(7) $$\vec{g_{m}}=(sin\beta_{m}cos\alpha_{m},sin\beta_{m}sin\alpha_{m},cos\beta_{m})$$

Considering the aforementioned mapping model, the following functional relationship can be established:

(8) $$\left(\begin{array}{cccc} y_{m} & 1 & -b_{y} & 0\\ x_{m} & 0 & -b_{x} & 1 \end{array}\right)\left(\begin{array}{c} \frac{1}{kr}\\ cos\beta_{0}\\ \frac{1}{kr}\\ sin\beta_{0}sin\beta_{0} \end{array}\right)=\left(\begin{array}{c} cos\beta_{m}\\ sin\alpha_{m}sin\beta_{m} \end{array}\right)$$

According to solid geometry, the spatial coordinates of the gaze vector can be calculated as follows:

(9) $$\left\{\begin{array}{l} cos\beta_{m}=\frac{y(G_{m})}{\sqrt{{\left[x(G_{m})-\frac{d}{2}\right]}^{2}+y{\left(G_{m}\right)}^{2}+z{\left(G_{m}\right)}^{2}}}\\ sin\beta_{m}sin\alpha_{m}=\frac{x(G_{m})-\frac{d}{2}}{\sqrt{{\left[x(G_{m})-\frac{d}{2}\right]}^{2}+y{\left(G_{m}\right)}^{2}+z{\left(G_{m}\right)}^{2}}}\\ sin\beta_{m}cos\alpha_{m}=\sqrt{1-{\left(sin\beta_{m}sin\alpha_{m}\right)}^{2}-{\left(cos\beta_{m}\right)}^{2}} \end{array}\right.$$

This system establishes *N* spatial points that are predetermined at specific positions for the purpose of parameter calibration. Clearly, the parameter vector can only be calibrated if *N* is greater than or equal to 2. In practical applications, due to errors in line-of-sight estimation, it is essential to collect multiple data sets and construct the matrix equation A*x* = B to approximate the solutions for the system parameters. Here, *x* represents the vector of the system parameters to be determined, while A and B are defined as follows:

(10) $$\left\{A={\left[\begin{array}{cccc} y_{1} & 1 & -b_{y} & 0\\ x_{1} & 0 & -b_{x} & 1\\ & \ldots & & \\ y_{N} & 1 & -b_{y} & 0\\ x_{N} & 0 & -b_{x} & 1 \end{array}\right]}_{2N\times 3},B={\left[\begin{array}{c} cos\beta_{1}\\ sin\alpha_{1}sin\beta_{1}\\ \ldots \\ cos\beta_{N}\\ sin\alpha_{N}sin\beta_{N} \end{array}\right]}_{2N\times 1}\right.$$

In this context, the previously mentioned matrix forms a system of overdetermined equations. To address this system, the least squares method is employed as the primary approach, utilizing singular value decomposition to minimize ||A*x* − B||. This process allows the model to optimize performance at each estimation point. The algebraic expression representing this is as follows:

(11) $$\left\{\begin{array}{c} A=U\Sigma V\\ x=V\Sigma^{+}V^{T}B \end{array}\right.$$

**Figure 8:**
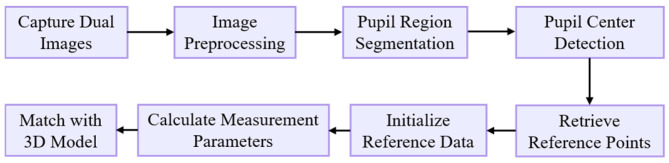
Gaze tracking system algorithm flow.

The matrices *U*, Σ, and *V* are derived from the singular value decomposition of matrix A, where Σ^+^ denotes the pseudoinverse of Σ. The parameter vectors for the left and right eyes can be obtained using the aforementioned methods. When the user looks in any direction, the system captures binocular images in real time. By extracting data from these binocular images and substituting it into the previously described algorithm, the spatial gaze point can be estimated. The complete algorithmic process is illustrated in Figure 8.

## 4. Experimental Results and Analysis

A total of 30 healthy adult Asian subjects, consisting of 15 male and 15 female participants, were recruited and involved in the experimental tests. They participated in laboratory-based wear trials to systematically evaluate the robustness of the proposed algorithm. Participants met the inclusion criteria of having normal or corrected-to-normal vision, no ocular diseases, no severe head tremor, and no history of eye surgery, while those with strabismus, neurological disorders, or inability to maintain stable head posture were excluded. Among them, 12 wore glasses, 5 wore contact lenses, and 13 had uncorrected vision; eye dominance was balanced (16 right-eye dominant, 14 left-eye dominant), with an average interpupillary distance of 63.5 ± 2.1 mm.

**Figure 9:**
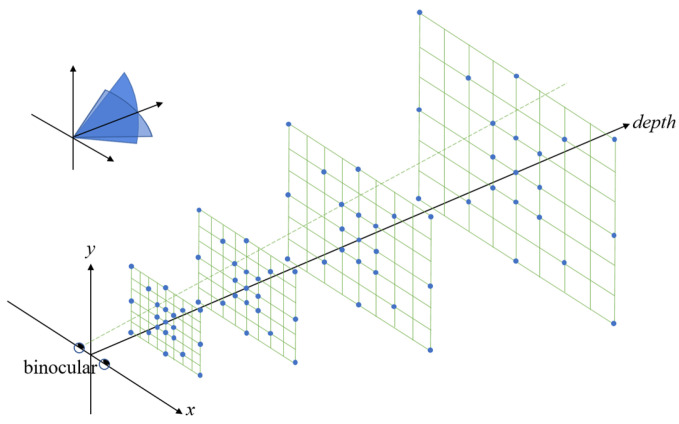
The truth distribution of spatial gaze points. The coordinate system in the top-left corner illustrates the horizontal and vertical field-of-view ranges of the gaze. In the main diagram, blue dots denote gaze points distributed on each depth plane, while the green grids depict the relative positional relationships among these gaze points. Blue dots connected by dashed lines correspond to gaze points at the same field-of-view angle across different depths.

Experiments were conducted at seven fixed depth planes which were evenly spaced within the range of 0.3 m to 1 m. To ensure a rigorous and unbiased evaluation, calibration and test points were strictly separated and never reused across phases. As detailed in Section 3.6, a fixed set of 19 calibration points was used to estimate the model parameters, while a disjoint set of 15 test points was used for accuracy evaluation at each depth plane. No test point was included in the calibration set, eliminating any risk of inflated accuracy due to data leakage. At each plane, participants completed 15 test targets, with five repetitions per test target. All tests were performed in a controlled indoor environment with uniform soft lighting, and participants rested their chin on a soft support to maintain natural head posture and minimize movement. The head-mounted device integrated an industrial-grade 950 nm infrared camera as the input sensor, paired with an IR LED fill light of identical central wavelength, capturing video at 1920 × 1080 resolution and 60 FPS. The software environment included Python 3.12, and OpenCV 4.8. During each test, they were instructed to fixate on a target point displayed on the screen. We conducted a comparative experiment on the mainstream laptop hardware platform, which was equipped with an Intel Core Ultra 9 288V processor. The base frequency was 2.20 GHz, it had eight cores and eight threads, and the single-core turbo frequency could reach 5.10 GHz. It adopted the Lunar Lake architecture, with a 3 nm manufacturing process and a TDP power consumption of 30 W, and the memory used 32 GB LPDDR5X RAM. A commercial high-precision 3D eye tracker (RED 500, SensoMotoric Instruments GmbH, Teltow, Germany) was adopted as the ground truth reference, which can provide accurate 3D point of regard coordinates with a mean error of less than 0.5 mm [22]. Regarding recalibration frequency, a one-time calibration procedure was performed at the beginning of each participant’s session. Considering that each participant spent only a short time on the experiment, the calibrated parameters were used consistently for all subsequent depth planes and test trials. The calibrated parameters were used consistently for all subsequent depth planes and test trials. This design choice was validated in preliminary experiments, which confirmed stable performance across the 0.3–1.0 m depth range without recalibration. We evaluated the 2D angular error and the absolute 3D Euclidean position error across the seven depth planes, to fully characterize the 3D behavior of the proposed method; the required spatial coordinate points for observation are shown in Figure 9.

**Figure 10:**
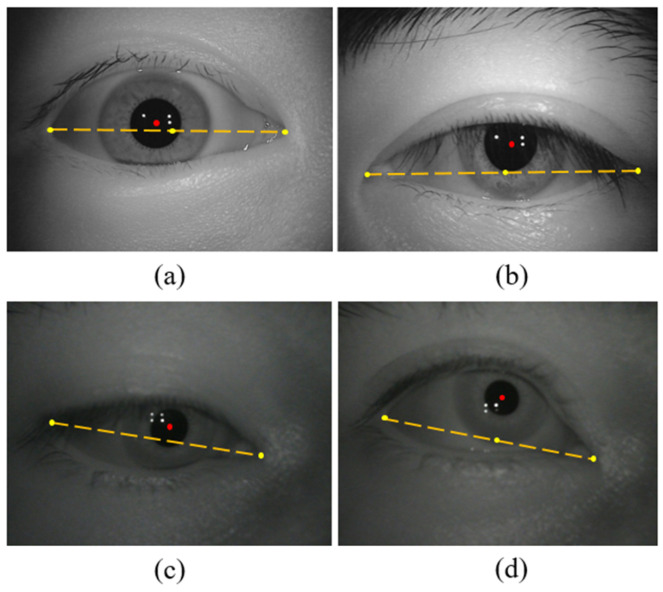
Detected pupil center images. In each subfigure, the red dot denotes the pupil center estimated by the proposed method. The leftmost and rightmost yellow points mark the outer and inner canthus corners, respectively. The dashed line is the line connecting the two canthus corners, and the middle yellow point is the midpoint of this line. (**a**) Ideal state. (**b**) Partially obscured. (**c**) Eyelash interference. (**d**) Extreme observation perspective.

During human eye observation, several highly disruptive conditions are unavoidable. Specifically, optical imaging effects can induce non-linear pupil deformation, such as stretching and squeezing, when the eye views objects from extreme angles. Additionally, partial occlusion of the pupil may occur, which compromises pupil integrity and presents a substantial challenge to accurate feature extraction and localization. Figure 10 illustrates representative pupil center detection results for these scenarios, where the red dot marks the detected pupil center, and the three yellow dots and the dotted line represent the medial and lateral canthi and the midpoint of the line connecting them. Even at extreme viewing angles, where the pupil deviates from its ideal circular shape to an irregular elliptical form, the proposed algorithm reliably and accurately segments and identifies the elliptical pupil region. In addition, for test cases where pupil occlusion does not exceed 40%, the algorithm effectively mitigates interference through contextual feature completion and redundant feature verification, consistently producing stable localization results that match the true pupil position without failure due to partial information loss.

**Figure 11:**
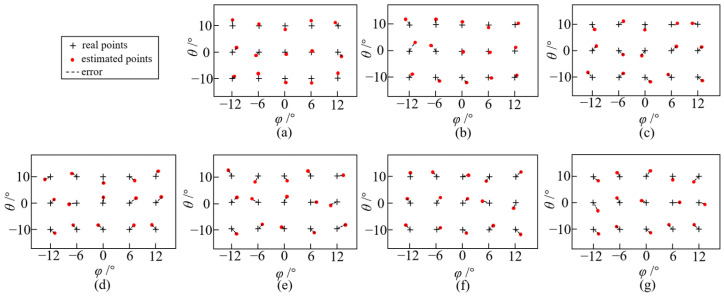

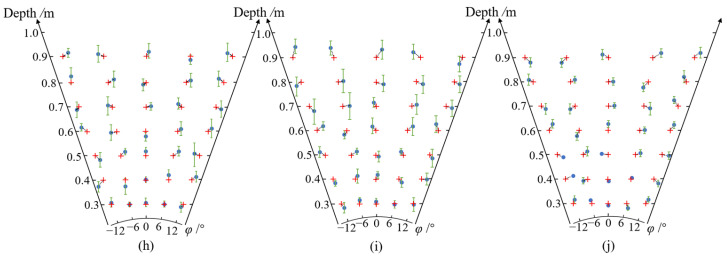
Gaze estimation performance across horizontal (φ) and vertical (θ) viewing angles, and seven depth planes. 2D scatter plots comparing ground truth (black plus signs) and estimated (red dots) gaze directions at (**a**) 0.3 m, (**b**) 0.4 m, (**c**) 0.5 m, (**d**) 0.6 m, (**e**) 0.7 m, (**f**) 0.8 m, and (**g**) 0.9 m depth plane, covering horizontal gaze angles φ ∈ [−12°, 12°] and vertical gaze angles θ ∈ [−10°, 10°], with dashed lines representing the estimation error. 3D visualization plots illustrating the 3D gaze estimation results across the horizontal gaze angle axis (φ), depth axis, for the (**h**) upward gaze angle (θ = 10°), (**i**) downward gaze angle (θ = −10°), and (**j**) neutral gaze angle (θ = 0°), where the red plus signs indicate the ground truth 3D points, the blue dots represent the estimated 3D points, and the green error bars denote the 95% confidence intervals of depth estimation derived from multiple fixations on the same target point.

In a spherical coordinate system with the eyeball as the origin, gaze movement within a range of ±12° vertically and ±10° horizontally leads to the experimental results presented in Figure 11.

**Figure 12:**
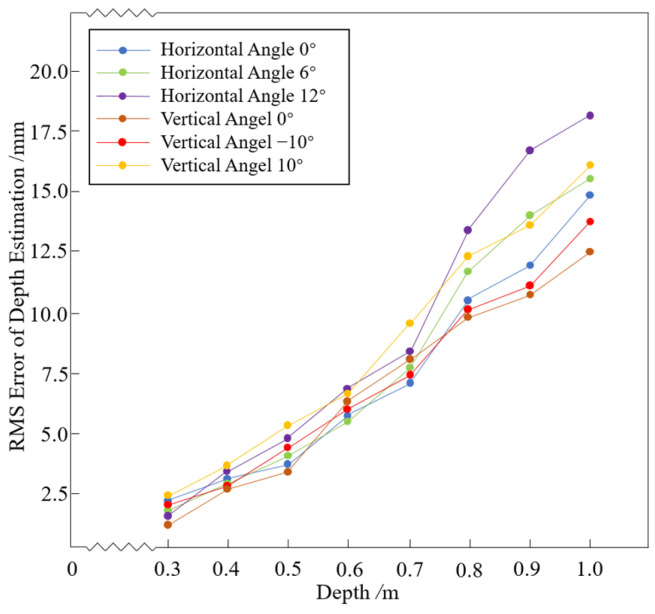
The distribution of RMS error of gaze point estimation varying with depth under different perspectives.

As shown in Figure 11, we first evaluated the angular estimation performance across all seven depth planes. In Figure 11a–g sub-figures, the black plus signs represent the ground truth gaze angles, the red dots represent the estimated gaze angles from the proposed method, and the black dashed lines connect the corresponding points to visualize the estimation error. The results clearly show that the estimated points are highly consistent with the ground truth points across all depth planes. For the 3D positioning performance, we further evaluated the estimation results under three different pitch conditions: upward gaze (θ = 10°), neutral gaze (θ = 0°), and downward gaze (θ = −10°), as shown in Figure 11h, Figure 11i and Figure 11j, respectively. In these 3D visualization plots, the horizontal axis denotes the horizontal gaze angle φ, and the oblique axis represents the target depth. The red plus signs indicate the ground truth 3D points, the green dots represent the estimated 3D points, and the error bars overlaid on the green dots denote the 95% confidence intervals of the depth estimation, derived from multiple fixations on the same target point. The confidence interval of the depth estimation remains small across all depth ranges, which means the proposed method can provide reliable depth estimation with stable uncertainty, even for far targets. We adopted the RMS error of three-dimensional coordinates to quantitatively assess the 3D positioning accuracy for each target point. The RMS error is calculated as

(12) $$RMS_{3D}=\sqrt{\frac{1}{N}\sum_{i=1}^{N}[{\left({\hat{x}}_{i}-x_{i}\right)}^{2}+{\left({\hat{y}}_{i}-y_{i}\right)}^{2}+{\left({\hat{z}}_{i}-z_{i}\right)}^{2}]}$$

where (*x_i_*, *y_i_*, *z_i_*) and (${\hat{x}}_{i},{\hat{y}}_{i},{\hat{z}}_{i}$) denote the ground truth and estimated 3D coordinates of each individual target point, respectively, and *N* is the total number of test samples. Figure 12 shows the distribution of the RMS of the gaze point estimation error varying with depth under different perspectives.

As illustrated in Figure 12, the RMS error of depth estimation increases monotonically with target distance for all gaze directions, following a sub-quadratic growth trend that reflects the inherent uncertainty of stereo triangulation. At 0.3 m, the error remains below 3 mm for all configurations; at 1.0 m, it rises to approximately 18 mm under the most challenging ±12° horizontal condition. The most significant performance degradation occurs with increasing horizontal gaze angles: errors at ±6° and ±12° are substantially higher than at 0°, resulting in an overall RMS error of 2.82° for the proposed method. In contrast, vertical gaze angles (±10°) cause only a modest increase in error, with the upper and lower curves nearly indistinguishable. Overall, the proposed system maintains RMS depth accuracy within 20 mm across the intended 0.3–1.0 m working range, even for large off-axis angles, ensuring reliable performance for typical laptop and near-eye interaction scenarios. As the target distance increases from 30 cm to 90 cm, the 3D position error increases from 8.23 mm to 18.92 mm, with a slight increase in angular error. This trend follows the inherent principle of binocular stereo vision. We analyzed the correlation between the estimated vergence angle, which is the angle between the left and right eye gaze vectors, and the true target distance, which is the core physiological cue for human depth perception. The results show that the correlation coefficient between the estimated vergence angle and the true target distance reaches 0.97 overall, and remains above 0.95 across all seven depth planes. This indicates that as the target distance changes, the estimated vergence angle changes synchronously and stably, which proves that the proposed method can truly capture the 3D characteristic of the gaze.

We further compared the proposed method with state-of-the-art existing methods, including academic solutions based on pupil core eye-tracking goggles [23], and the academic method for generating accurate 3D gaze vectors using synchronized eye tracking and motion capture [24], to clarify the contribution of the proposed method. The results are summarized in Table 2.

Table 2 compares the performance of our proposed method with two state-of-the-art gaze tracking approaches. The proposed lightweight geometric method achieves comparable angular error (1.1–2.82°) to the other two methods, while it significantly outperforms them in terms of 3D position error. More importantly, our approach provides a competitive 3D position RMS error of less than 13.24 mm, which is 34% lower than the eye-motion capture-based method (<20 mm) and 56% lower than the noise estimation-based method (<30 mm). This balance of high angular precision and low 3D positioning error validates the effectiveness of our self-developed geometric framework for practical eye-tracking applications. We further include three representative consumer-grade eye-tracking systems, namely Apple Vision Pro with M5, VIVE Focus Vision, and Windows Studio, as detailed in Table 3.

It should be noted that these performance values are taken from official specifications and published materials, and were not evaluated under the same controlled experimental conditions as our method. While these commercial platforms may achieve comparable angular accuracy in their intended use cases, they typically do not report detailed 3D position error metrics. In contrast, our approach delivers both competitive angular error and explicitly validated superior 3D positioning precision, demonstrating its suitability for high-precision 3D gaze tracking applications. Compared with high-end commercial systems, the proposed method achieves comparable 3D positioning accuracy with much lower hardware cost, without requiring dedicated and costly high-precision sensors, making it suitable for entry-level devices.

## 5. Conclusions

This study presents a lightweight, purely geometric framework for high-precision 3D gaze tracking based on infrared image processing, which achieves robust and real-time performance on low-cost, resource-constrained platforms. The proposed method abandons heavy deep learning networks and relies on geometric modeling, binocular stereo constraints, and infrared optical design to accurately estimate 3D gaze under variable viewing angles and depth conditions, effectively suppressing performance degradation caused by large viewing angles, pupil occlusion, and unreliable depth extraction in low-resolution infrared images. Experimental results show that the framework achieves favorable precision, strong robustness against partial occlusion, as well as extremely low computational overhead and high real-time performance. Compared with appearance-based deep learning methods, conventional model-based schemes and commercial eye-tracking devices, the proposed framework exhibits clear advantages in terms of computational expense, ease of deployment, 3D estimation accuracy and costs, making it suitable for head-mounted devices, wearable systems, automotive driver monitoring, and portable human–computer interaction applications. Future work will focus on further improving robustness under extreme head rotations, long-distance measurement, and eyeglass interference, as well as optimizing the geometric model to further improve the accuracy of gaze estimation and extend the method to more compact binocular infrared sensing architectures.

## Figures and Tables

**Table 1:** Comparison of pupil center localization methods.

Algorithm	Key Parameters	Accuracy/%
Gradient-based [20]	Sobel kernel 3 × 3, edge threshold = 30	85.9
Hough transform	Edge threshold = 30, min ellipse area = 150 pixels	84.1
Neural network [21]	Input size 128 × 128, batch size = 3250 training epochs	82.2
Proposed method in this paper	See Section 3.4 for detailed implementation	92.5

**Table 2:** Performance comparison of the proposed method with latest academic systems.

Method	Type	Angular Error (°)	3D Position Error (mm)
Noise estimation-based [23]	Academic	1.5–2.7°	<30
Eye-motion capture-based [24]	Academic	1–2°	<20
Proposed	Academic	1.1–2.82°	<13.24

**Table 3:** Performance specifications of representative commercial eye-tracking devices.

Method	Type	Angular Error (°)	3D Position Error (mm)
Apple Vision Pro with M5 [14]	Commercial	0.5°~1.0°	-
VIVE Focus Vision [15]	Commercial	0.5°~1.1°	-
Windows Studio [16]	Commercial	1.2°	10

## Data Availability

The original contributions presented in this study are included in the article. Further inquiries can be directed to the corresponding author.
